# Open-source, partially 3D-printed, high-pressure (50-bar) liquid-nitrogen-cooled parahydrogen generator

**DOI:** 10.5194/mr-2-49-2021

**Published:** 2021-02-18

**Authors:** Frowin Ellermann, Andrey Pravdivtsev, Jan-Bernd Hövener

**Affiliations:** Section for Biomedical Imaging, Molecular Imaging North Competence Center (MOIN CC), Department of Radiology and Neuroradiology, University Medical Center Schleswig-Holstein (UKSH), Kiel University, Kiel 24118, Germany

## Abstract

The signal of magnetic resonance imaging (MRI) can be enhanced by several orders of magnitude using hyperpolarization. In comparison to a broadly used dynamic nuclear polarization (DNP) technique that is already used in clinical trials, the parahydrogen ( p H ${}_{2})$ -based hyperpolarization approaches are less cost-intensive, are scalable, and offer high throughput. However, a p H ${}_{2}$ generator is necessary. Available commercial p H ${}_{2}$ generators are relatively expensive (EUR 10 000–150 000). To facilitate the spread of p H ${}_{2}$ -based hyperpolarization studies, here we provide the blueprints and 3D models as open-source for a low-cost (EUR <3000 ) 50-bar liquid-nitrogen-cooled p H ${}_{2}$ generator.

## 1 Introduction

Nuclear magnetic resonance (NMR) as well as magnetic resonance imaging (MRI) are widely used in medical imaging and chemical analysis. Despite the great success of these techniques (Feyter et al., 2018; Lange et al., 2008; Watson et al., 2020), the low signal-to-noise ratio of NMR limits promising applications such as in vivo spectroscopy or imaging of nuclei other than ${}^{1}$ H (Wilferth et al., 2020; Xu et al., 2008). The hyperpolarization of nuclear spins boosts the signal of selected molecules by orders of magnitude. This way, imaging of the lung or metabolism has become feasible (Beek et al., 2004; Kurhanewicz et al., 2011).

Among techniques, parahydrogen and synthesis allows dramatically enhanced nuclear alignment (PASADENA) (Bowers and Weitekamp, 1986, 1987; Eisenschmid et al., 1987) has found application from catalysis research to metabolic imaging (Hövener et al., 2018; Kovtunov et al., 2018).

The production of parahydrogen ( p H ${}_{2})$ is relatively easy: H ${}_{2}$ gas flows through a catalyst at cold temperatures; maximum para-enrichment of almost 100 % is achieved at about 25 K (Gamliel et al., 2010; Jeong et al., 2018; Kiryutin et al., 2017). To reach low temperatures, hence enriched p H ${}_{2}$ , liquid cryogens (Buckenmaier et al., 2018; Jeong et al., 2018) or electric cryopumps (Feng et al., 2012) are used. Electronic setups were reported, e.g. for pressures up to 50 bar of ≈100 % p H ${}_{2}$ (Hövener et al., 2013). Liquid nitrogen (lN ${}_{2})$ -based systems were presented, however often with limited description, low production rate, and pressure.

Thus, in this contribution, we report a parahydrogen generator (PHG) based on lN ${}_{2}$ that operates at a pressure of up to 50 bar at a cost of less than EUR 3000. The setup is easy to replicate as it is fully open source (Ellermann, 2020b) and all parts are either off-the-shelf or 3D-printed or can be constructed easily. Besides, we introduce an automated p H ${}_{2}$ quantification method using a 1 T benchtop NMR and a microcontroller-based process control (Arduino).

### 1.1 Background

In 1933 Werner Heisenberg received his Nobel Prize “for the creation of quantum mechanics, the application of which has, inter alia, led to the discovery of the allotropic forms of hydrogen” (NobelPrize.org, 2020). Allotropy is a property of substances to exist in several forms in the same physical state. Two forms of hydrogen usually are referred to as nuclear spin isomers; they are parahydrogen ( p H ${}_{2})$ and orthohydrogen ( o H ${}_{2})$ . Hydrogen is not the only compound that has stable or long-lived spin isomers at room temperature (rt); there are many examples: deuterium (Knopp et al., 2003), water (Kravchuk et al., 2011; Vermette et al., 2019), ethylene (Zhivonitko et al., 2013), and methyl groups (Meier et al., 2013). Although some molecules are not symmetric and cannot be extracted at room temperature, they possess long-lived spin states of minutes (Pileio et al., 2008) and hours (Stevanato et al., 2015).

**Figure 1:**
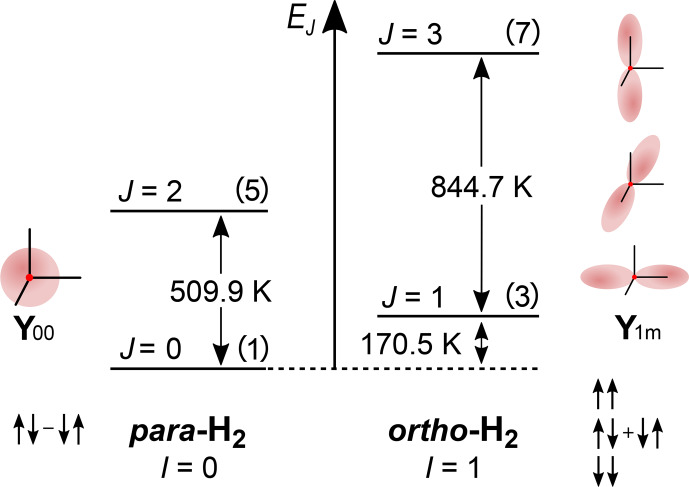
The rotational energy level diagram for isolated H ${}_{2}$ . The angular distribution of the two lowest rotational states ( $Y_{00}$ corresponds to J=0 , and $Y_{1+1}$ , $Y_{1-1}$ and $Y_{10}$ correspond to J=1) and spin states of orthohydrogen and parahydrogen are indicated. The numbers in parentheses are the degeneracies of the state 2J+1 . The energy of rotation spin states in units of K is equal to $E_{J}=J\left(J+1\right)\theta_{R}$ with $\theta_{R}=87.6\;K$ (Atkins and De Paula, 2006). The distance between two adjacent energy levels is $E_{J+1}-E_{J}=2\left(J+1\right)\theta_{R}$ . The figure was inspired by an illustration of I. F. Silvera (1980).

The spin of hydrogen nuclei (of protons) is the origin of the two nuclear spin-isomer forms of dihydrogen. Protons have spin-1/2; hence, they are fermions. Fermions are particles that follow the Fermi–Dirac statistics; therefore, the sign of the total wave function of H ${}_{2}$ has to change when two nuclei are exchanged. The spin space of two spin-1/2 consists of ${\left(2\cdot \frac{1}{2}+1\right)}^{2}=4$ states. They are three symmetric spin states, $\left|T_{+}\right.\rangle =\left|\alpha \alpha \right.\rangle$ , $\left|T_{0}\right.\rangle =\left(\left|\alpha \beta \right.\rangle +\left|\beta \alpha \right.\rangle \right)/\sqrt{2}$ , $\left|T_{-}\right.\rangle =\left|\beta \beta \right.\rangle$ , and one asymmetric nuclear spin state, $\left|S\right.\rangle =\left(\left|\alpha \beta \right.\rangle -\left|\beta \alpha \right.\rangle \right)/\sqrt{2}$ (Fig. 1). Here, conventionally $\left|\alpha \right.\rangle$ and $\left|\beta \right.\rangle$ states are nuclei spin states with the projection of spin on the Z axis 1/2 and -1/2 , $\left|T_{+}\right.\rangle$ , $\left|T_{0}\right.\rangle$ , $\left|T_{-}\right.\rangle$ are triplet spin states of two spin-1/2 with a total spin of 1 and the projection on the Z axis +1 , 0, and -1 , and $\left|S\right.\rangle$ is a singlet spin state of two spin-1/2 with a total spin of 0.

The rotational wave function after nuclei permutation does not change, because of the molecular symmetry, and is only multiplied by ${\left(-1\right)}^{J}$ , with J being the rotational quantum number of the state. Hence, H ${}_{2}$ with a symmetric nuclear spin state (triplet states) can only have an asymmetric rotational state ( J is odd); such H ${}_{2}$ is called o H ${}_{2}$ , and vice versa, H ${}_{2}$ with an asymmetric nuclear spin state (singlet state) can only have symmetric rotation states ( J is even); such H ${}_{2}$ is called p H ${}_{2}$ .

**Figure 2:**
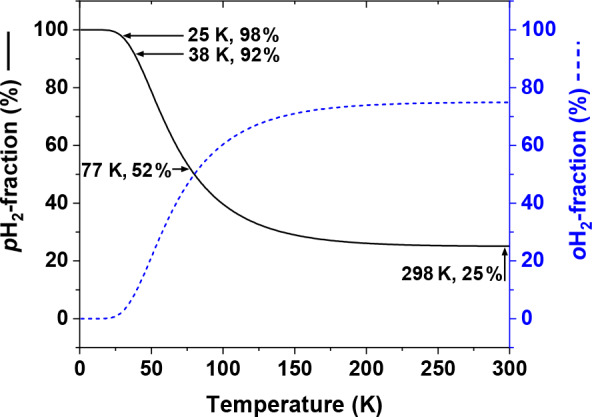
Thermal equilibrium fractions of p H ${}_{2}$ and o H ${}_{2}$ as a function of temperature calculated with Eq. (1) and J=6 . Four temperatures are marked: (1) 298 K – “room temperature”, $f_{pH_{2}}≅25\;\%$ , (2) 77 K – the boiling temperature of liquid nitrogen, $f_{pH_{2}}≅52\;\%$ , (3) 38 K – medium conversion temperature of the Bruker p H ${}_{2}$ generator, $f_{pH_{2}}≅92\;\%$ , and (4) 25 K – conversion temperature of high-pressure PHG, $f_{pH_{2}}≅98\pm 2\;\%$ (Hövener et al., 2013).

The difference in the energy levels of two ground states of ortho ( J=1) and para ( J=0) hydrogen is $E_{J=1}-E_{J=0}=2\theta_{R}≅175\;K$ (Fig. 1) (Atkins and De Paula, 2006). Such a big energy gap allows a relatively simple way of spin-isomer enrichment: for H ${}_{2}$ the ground state is p H ${}_{2}$ and its population can be increased by cooling down the gas (Fig. 2) (M. Richardson et al., 2018). The ratio of the number of molecules of p H ${}_{2}$ , $n_{pH_{2}}$ , to o H ${}_{2}$ , $n_{oH_{2}}$ , in thermal equilibrium is given by the Boltzmann distribution of rotational energy levels:

1 $$\frac{n_{pH_{2}}}{n_{oH_{2}}}=\frac{\sum_{J=\mathrm{even}\geq 0}\left(2J+1\right)\mathrm{exp}\left(-J\left(J+1\right)\theta_{R}/T\right)}{3\sum_{J=\mathrm{odd}\geq 1}\left(2J+1\right)\mathrm{exp}\left(-J\left(J+1\right)\theta_{R}/T\right)}.$$

Since only two nuclear spin-isomer states of H ${}_{2}$ exist, their fractions can be easily obtained: $f_{pH_{2}}=\frac{n_{pH_{2}}}{n_{pH_{2}}+n_{oH_{2}}}=\frac{1}{1+\frac{n_{oH_{2}}}{n_{pH_{2}}}}$ and $f_{oH_{2}}=\frac{1}{1+\frac{n_{pH_{2}}}{n_{oH_{2}}}}$ . At room temperatures ( T≅298K) $n_{pH_{2}}\;:\;n_{oH_{2}}$ is close to 1:3 , at 77 K – the normal boiling point of nitrogen – the ratio is close to 1:1 , and at 25 K $f_{pH_{2}}≅98\;\%$ (Fig. 2).

**Table 1:** Performance comparison of several PHGs: (1) Bruker PHG 90, (2) dual-stage cryostats (DSC) (Hövener et al., 2013), (3) (Feng et al., 2012), (4) HyperSpin-PHG (Meier et al., 2019), (5) automated PHG (Birchall et al., 2020), (6) He-dewar PHG (Du et al., 2020), (7) U-shaped PHG (Kiryutin et al., 2017), (8) economical PHG (Jeong et al., 2018), (9) glass-trap PHG (Gamliel et al., 2010), and (10) in-house-designed and -built PHG (this work). Given prices include all connectors, cylinders, and 19 % VAT. lN ${}_{2}$ stands for liquid nitrogen and “cc-He” for closed-cycle He compressor.

No.	Name	Operating temperature	$f_{pH_{2}}(\%)$	Flow rate (SLM)	Max. pressure (bar)	Price (EUR)
		(K) (method)				
1	Bruker PHG 90	36–40 [cc-He]	85-92	≤0.2	10	100 000–150 000
2	Continuous DSC PHG (Hövener et al., 2013)	25 [cc-He]	98±2	4	50	37 000
3	Pulsed DSC PHG (Feng et al., 2012)	15 [cc-He]	98	0.9	20	N.A.
4	HyperSpin-PHG (Meier et al., 2019)	20–77 [cc-He]	N.A. ${}^{a}$	N.A.	Min. 10	N.A.
5	Automated PHG (Birchall et al., 2020)	40 [cc-He]	∼87	0.15	33.8	<25 000
6	He-dewar PHG (Du et al., 2020)	30 [He]	97.3±1.9	∼0.3	4.5	N.A.
7	U-shaped PHG (Kiryutin et al., 2017)	77 [lN ${}_{2}$ ]	∼50	0.36 ${}^{b}$	Min. 3	N.A.
8	Economical PHG (Jeong et al., 2018)	77 [lN ${}_{2}$ ]	∼50	N.A.	N.A.	N.A.
9	Glass-trap PHG (Gamliel et al., 2010)	77 [lN ${}_{2}$ ]	46.3±1.3	0.0025 ${}^{c}$	∼1	N.A.
10	This work	77 [lN ${}_{2}$ ]	51.6±0.9	2.0 ${}^{d}$	50 ${}^{e}$	2988 ${}^{f}$

${}^{a}$ Depends on choice of coolant. ${}^{b}$ Estimated average flow (3.5 L volume filled to 3 bar in 90 min) calculated by us. ${}^{c}$ Estimated average flow (0.6 L volume filled to 1 bar in 240 min) calculated by us. ${}^{d}$ Highest average flow on filling 1 L bottle to 10 bar without sacrificing enrichment. ${}^{e}$ 50-bar p H ${}_{2}$ delivery was tested. Used parts allow pressure of at least 100 bar (safety margin). ${}^{f}$ Including H ${}_{2}$ -gas sensor and excluding H ${}_{2}$ and N ${}_{2}$ bottles/regulators.

### 1.2 Technology review

p H ${}_{2}$ fraction, $f_{pH_{2}}$ , of 90 % and above is produced by PHGs with single- or dual-stage cryostats run by helium compressors. A single-stage cryostat was reported to operate at 36–40 K with a flow rate of 0.2 SLM (standard litres per minute), 10-bar maximum delivery pressure, and $f_{pH_{2}}≅85$ %–92 % (Bruker, Billerica, USA); dual-stage cryostats operate at temperatures below 25 K, where $f_{pH_{2}}$ reaches 100 % (note that the boiling point of H ${}_{2}$ is 21 K) (Haynes, 2011). All these PHGs were specifically designed with PHIP (parahydrogen-induced polarization) in mind, meaning for a relatively low scale of production and in-lab use (not for industry). These setups required some on-site assembly and were realized in different designs, e.g. with pulsed injection (Feng et al., 2012) or continuous flow (Hövener et al., 2013). The continuous flow setup was reported to operate at a conversion temperature of 25 K, a 4 SLM flow rate, a 50-bar maximum delivery pressure, and an experimentally obtained $f_{pH_{2}}≅98\pm \;2\;\%$ (Hövener et al., 2013).

These setups work reliably and do not require the supply of liquid cryogens. Disadvantages, however, include high initial investments (EUR 40 000–150 000), some maintenance of the He compressor and cryostat ( ≈ EUR 10 000 every 25 000 h operational time), some site requirements ( ∼4 kW cooling water, appropriate safety precautions), and operational cost in the form of electricity ( >4 kW electrical power) (Table 1).

A 100 % p H ${}_{2}$ enrichment, however, may not always be needed: 50 % p H ${}_{2}$ fraction already provides one-third of the maximum polarization at one-tenth of the cost (or less) (M. Richardson et al., 2018). To achieve $f_{pH_{2}}$ of 50 %, 77 K, the temperature of lN ${}_{2}$ , is sufficient. Indeed, lN ${}_{2}$ -based PHGs are still used in many studies (Kiryutin et al., 2017; Meier et al., 2019). The design of such PHGs is generally simple – a catalyst chamber or tube immersed in lN ${}_{2}$ , but just like cryostat-based PHGs, lN ${}_{2}$ -based PHGs are continuously improving. As such, recent advances included a remarkable work where 20 L lN ${}_{2}$ was sufficient to provide p H ${}_{2}$ continuously for 20 d (Jeong et al., 2018).

Interestingly, in various cases it was demonstrated that an increased flow rate and pressure of p H ${}_{2}$ can boost the signal of PHIP or signal amplification by reversible exchange (SABRE) (Adams et al., 2009; Rayner and Duckett, 2018) beyond the factor of 3 offered by PHGs, with close to $f_{pH_{2}}≅100\;\%$ (Colell et al., 2017; Rayner et al., 2017; Štěpánek et al., 2019; Truong et al., 2015).

## 2 Methods

### 2.1 3D design of PHG

**Figure 3:**
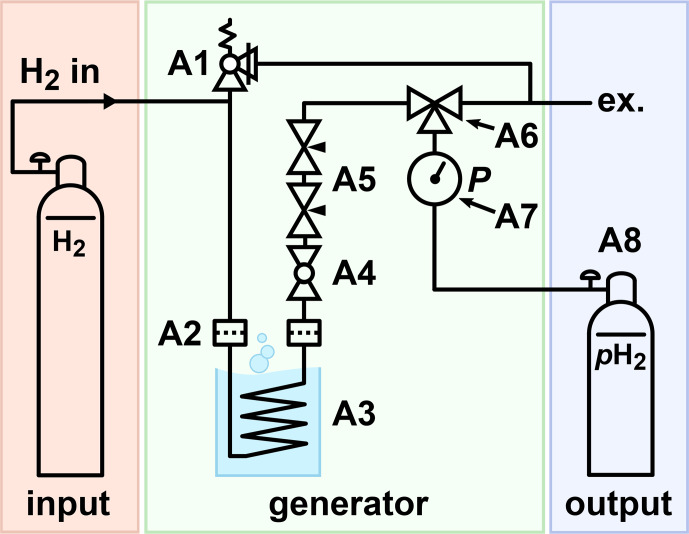
Schematic view of the PHG. H ${}_{2}$ gas was supplied via the inlet, e.g. from a 50 L 200-bar cylinder. The gas flowed through a filter (A2) into the ortho-para-conversion unit (A3) immersed in lN ${}_{2}$ , where it cooled down and thus was enriched with the p H ${}_{2}$ component. The parahydrogen-enriched gas exited the ortho-para-conversion unit, warmed up, and passed another particle filter. The filters reduced the contamination of the setup with the catalyst. A ball valve (A4) was used to start or stop the gas flow. Two needle valves (A5) were used to control the flow rate. A three-way valve (A6) allowed the storage cylinder to be filled or drained. A 100-bar safety valve (A1) was connected to the system to relieve a potential excess of pressure.

The principal scheme of the lN ${}_{2}$ -based PHG presented here consists of a H ${}_{2}$ gas supply, a generator, and a p H ${}_{2}$ storage (Fig. 3). A model of the PHG was designed (Autodesk Inventor 2019, San Rafael, USA). Aluminium profiles and steel angles (30 mm, Bosch Rexroth, Stuttgart, Germany) were used to construct the chassis. Copper tubes (outer diameter 6 mm, inner diameter 4 mm, rated for 229 bar, R220, Landefeld, Kassel-Industriepark, Germany) and valves (Swagelok, Solon, USA) were mounted on the chassis using 3D-printed parts (Ultimaker PLA “Perlweiss” Filament, Ultimaker S5, Ultimaker Cura, Utrecht, Netherlands). A 2 L stainless steel dewar was placed in the chassis (DSS 2000, 2 L, KGW Isotherm, Karlsruhe, Germany). The same copper tubes were used to wind a coil with 5.4 turns and a diameter of 86 mm. About 1.5 mL granular Fe(OH)O (371254-50G, Sigma-Aldrich, St. Louis, USA) was filled into the coil. On both ends of the copper coil, cotton wool was pressed to keep the catalyst in place to protect the rest of the system from contaminations. The compressed wool insets have a length of 20 mm. Wool as a particulate filter was used before in another PHG (Du et al., 2020). During the 6 months of weekly use of our generator, there was no sign of a moving catalyst. All fittings, T-pieces, ball valves, an overpressure valve, flow regulators, a pressure gauge, and fast connectors (Swagelok, Solon, USA) were connected with the same copper tube. For the storage of p H ${}_{2}$ , a 1 L cylinder made from aluminium was used (C1, A6341Q, Luxfer, Nottingham, UK). All parts were chosen to be rated for 100 bar or more to allow for a 100 % safety margin. A list of all parts is given in Appendix A. The models of the PHG, 3D-printing parts, and experimental macros (experimental protocols) are available (Ellermann, 2020b).

### 2.2 Safety concept

A crucial part of a PHG is the development of a safety concept which includes a detailed risk assessment and comprehensive operating manual. The handling of pressurized H ${}_{2}$ gas entails the risk of pressured gases, forming a potentially explosive mixture with air as well as hydrogen embrittlement (Beeson and Woods, 2003; National Aeronautics and Space Administration, 1997). To reduce these risks, the following safety requirements were set.

1. Safety by design
  a. Pressure ratings of parts
    i. All components in contact with pressurized gas are rated for a minimum of 100 bar
    ii. Mechanical pressure gauge
    iii. 100-bar safety valve for overpressure control
  b. Avoidance of formation of explosive H ${}_{2}$ –air mixture and potential ignition
    i. Reduction of H ${}_{2}$ in the system by minimizing the inner volume of the gas lines
    ii. No electrical components in the system
    iii. Avoidance of temperatures above flame point
    iv. Avoidance of inductive and static spark charges in the gas lines (due to conductive and groundable pipe material)
    v. High H ${}_{2}$ throughput and storage of p H ${}_{2}$ in small cylinders lead to a short operating time of PHG
  c. Easy maintenance due to the simple and open design concept
2. Safety by site and operation
  a. Strong ventilation in the installation site
  b. No public access
  c. Appropriate warning signs
  d. Usage by trained personnel according to manual only
  e. Use of safety goggles and safety gloves for the handling of lN ${}_{2}$

  f. H ${}_{2}$ sensor (for leakage alarm at 50 ppm H ${}_{2}$ level)
  g. Regular inspection and maintenance

### 2.3 Production protocol

All p H ${}_{2}$ batches were produced in the same manner (the indices in the brackets relate to Fig. 3).

1. Preparation
  - Set initial state: close valve A4, connect the generator with the output via A6 (“fill” position)
  - Open and set supply of hydrogen to the appropriate pressure
  - Connect storage bottle A8 to the output
  - Fill the dewar with lN ${}_{2}$ and close the lid to reduce evaporation
  - Wait for 20 min and set the flow with regulator A5
2. Flush storage bottle
  - Open valve A4 and wait until the pressure gauge shows 3 bar
  - Release gas from the storage cylinder by connecting the storage bottle to the exhaust via A6 (“venting position”)
  - Repeat the flushing steps three times
3. Production and storage of p H ${}_{2}$
  - Set valve A6 to “fill” position
  - Wait until the gauge shows the desired pressure
  - Close valve A4
4. Finishing production of p H ${}_{2}$
  - Close storage bottle (bottle valve)
  - Close H ${}_{2}$ supply
  - Set valve A6 to “vent” position to reduce pressure in the output line
  - Disconnect storage bottle from the output (fast connect adapters keep the line closed) and connect an evacuated bottle or a bottle with a low H ${}_{2}$ pressure
  - Set valve A6 to “close” position
  - Ensure that the PHG is left with 2–5 bar of H ${}_{2}$

### 2.4 Quantification

#### 2.4.1 Flow quantification

We refrained from including a flow meter in the setup to keep it simple and robust. Instead, we used the time $t^{p,V}$ needed to fill a cylinder of a given volume $V_{0}$ to a given pressure $p_{\mathrm{target}}$ to measure the average flow rate $f_{r}$ of the p H ${}_{2}$ production. The pressure $p_{\mathrm{out}}$ in the outlet of the PHG increases during production. To obtain SLM, we used the following equation:

2 $$f_{r}=\frac{V_{0}}{t^{p,V}}\;\left[\mathrm{SLM}\right]=\frac{p_{\mathrm{out}}V_{0}T_{\mathrm{stand}}}{T_{\mathrm{rt}}p_{\mathrm{stand}}}\cdot \frac{1}{t^{p,V}},$$

where $T_{\mathrm{rt}}$ is the temperature of the quantification experiment (here: 22 ${}^{\circ }$ C) and “stand” stands for standard pressure and temperature values ( $p_{\mathrm{stand}}=10^{5}$ Pa, $T_{\mathrm{stand}}=273.15$ K) (Nič et al., 2009). The measurement of $f_{r}$ is performed in a regime where $p_{\mathrm{out}}$ is linear as a function of time ( $t^{p,V})$ , and hence it coincides with the initial flow rate that is usually reported.

#### 2.4.2 Gas system

A medium-pressure 5 mm NMR tube (522-QPV-8, Wilmad-LabGlass, Vineland, USA) was used for the p H ${}_{2}$ quantification and a heavy wall 5 mm NMR tube (Wilmad-LabGlass, 522-PV-9) for experiments with magnetic field cycling (MFC). Each of these NMR tubes was equipped with input and output gas lines (1/16 ${}^{\prime \prime }$ polytetrafluoroethylene capillary (PTFE) with 0.023 ${}^{\prime \prime }$ inner diameter) by gluing them to the cap. The other end of these tubes was connected to a custom-made valve system. The pressure in the system was set by changing the reducers of the respective gases and the back-pressure valve in the gas system (P-785, P-787, Postnova). The inlet gas pressure was regulated to achieve a steady bubbling for the given back pressures of 2.8 bar and 6.9 bar. The valve system is controlled with a microcontroller (Arduino) which was linked to the spectrometer software synchronizing the gas supply, venting of the NMR tube, and data acquisition. Using this gas system, we supplied the NMR tube with N ${}_{2}$ (99.999 %, Air Liquide), H ${}_{2}$ (99.999 %, Air Liquide), or p H ${}_{2}$ .

#### 2.4.3 p H ${}_{2}$ quantification protocol

**Figure 4:**
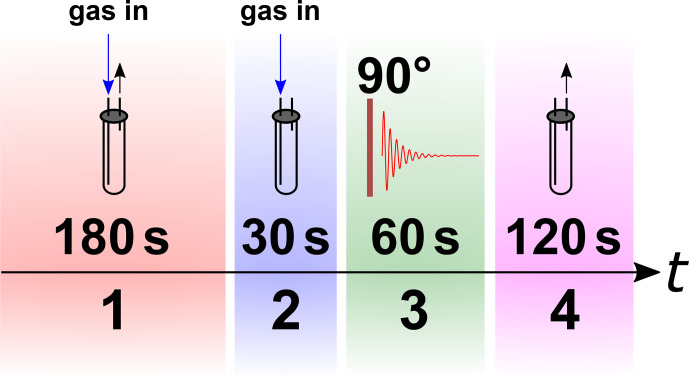
Scheme of the p H ${}_{2}$ quantification protocol. The NMR tube was flushed with N ${}_{2}$ , p H ${}_{2}$ , or rtH ${}_{2}$ gas for 180 s before the exhaust was closed. A rest time of 30 s was allowed for the system to settle down. Finally, the NMR spectra were acquired before the gas was released.

The p H ${}_{2}$ quantification was performed according to a quantification protocol (schematically shown in Fig. 4).

A NMR tube was placed in a 1 T NMR spectrometer (benchtop, SpinSolve Carbon 43 MHz, Magritek, Aachen, Germany) and not moved during the experiment. To remove air and residual gases from the lines, the setup was flushed with the gas for 3 min at a 5-bar input pressure and a fully open exhaust. Afterwards, the exhaust line was closed and a 30 s delay was allowed to stabilize pressure and flow before the NMR acquisition was started. To ensure constant pressure in the system, the gas supply was kept open during the NMR measurement. Because the NMR signal was not locked during the experiment, the H ${}_{2}$ resonance was moved to 0 ppm during post-processing for convenience.

All NMR spectra of gases were acquired with a standard excitation and acquisition pulse sequence (12.6 µ s excitation pulse that corresponds to 90 ${}^{\circ }$ flip angle, 20 ms acquisition time, 50 kHz spectral width, 0.5 s repetition time, 100 transients for averaging, SpinSolve Expert v3.54, Magritek, Aachen, Germany). The spectra were subjected to 20 Hz exponential apodization and phase correction. To remove background signals, a spectrum of N ${}_{2}$ was also acquired and subtracted from the rtH ${}_{2}$ (H ${}_{2}$ in thermal equilibrium at room temperature) and p H ${}_{2}$ spectra. After that, an automatic baseline correction (MNova v14.1.2, Santiago de Compostela, Spain) was applied to the phased spectrum. The spectral lines of rtH ${}_{2}$ and p H ${}_{2}$ were integrated within the borders of -15 and +15 ppm, giving S (rtH ${}_{2})$ and S(p H ${}_{2})$ . Finally the fraction of p H ${}_{2}$ $f_{pH_{2}}$ was calculated:

3 $$f_{pH_{2}}=\left(1-\frac{3}{4}\frac{S(pH_{2})}{S\left({\mathrm{rtH}}_{2}\right)}\right)\cdot 100\;\%.$$

Here we take into account that only o H ${}_{2}$ contributes to the MR signal and $f_{pH_{2}}=\frac{1}{4}$ at room temperature (Green et al., 2012).

### 2.5 SABRE experiment

#### 2.5.1 Sample preparation

The sample solution contained 3 mmol/L iridium N-heterocyclic carbene complex [Ir(COD)(IMes)Cl], where COD = 1,5-cyclooctadiene and Imes = 1,3-bis(2,4,6-trimethylphenyl) imidazol-2-ylidene (Cowley et al., 2011) and 26 mmol/L nicotinamide (CAS 98-92-0, Sigma-Aldrich) in methanol-d ${}_{4}$ 99.8 % (Deutero GmbH). To activate the catalyst, H ${}_{2}$ was flushed through the sample at 6.9 bar for 5 min before the magnetic field cycling experiments began.

#### 2.5.2 Magnetic field cycling experiment

**Figure 5:**
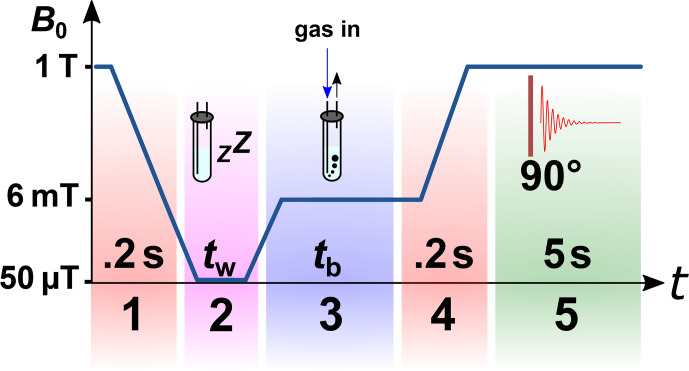
Scheme of the ${}^{1}$ H magnetic field cycling SABRE experiment. *Stage 1*: shuttling of the sample to the polarization coil. *Stage 2:* relaxation of the sample at earth's magnetic field for $t_{w}=10$ s. *Stage 3*: switching on the electromagnet with a magnetic field $B_{p}=6$ mT and starting bubbling with p H ${}_{2}$ -enriched gas at pressure P=6.9 bar or 2.8 bar for $t_{b}=30$ s. *Stage 4*: shuttling of the sample to the bore of the NMR spectrometer in 0.2 s and turning off the electromagnet. *Stage 5:* after 90 ${}^{\circ }$ excitation, acquiring the ${}^{1}$ H-NMR spectrum.

The NMR spectrometer was equipped with an in-house built MFC setup that will be described elsewhere. The shuttling time from the observation point to the homogenous area of the electromagnet was 0.2 s. The used electromagnet allowed a magnetic field variation in the range of -20 to +20 mT with a magnetic field homogeneity of 0.06 % in 2 cm. The same gas system as described above was used for the MFC SABRE experiments. The only modification was that a hollow optical fibre (Molex, part. no. 106815-0026, 250 µ m internal diameter, 360 µ m outer diameter) was glued to the end of the PTFE capillaries to reduce magnetic field distortions. All magnetic field cycling SABRE experiments were carried out according to the protocol in Fig. 5.

## 3 Results

### 3.1 PHG design

**Figure 6:**
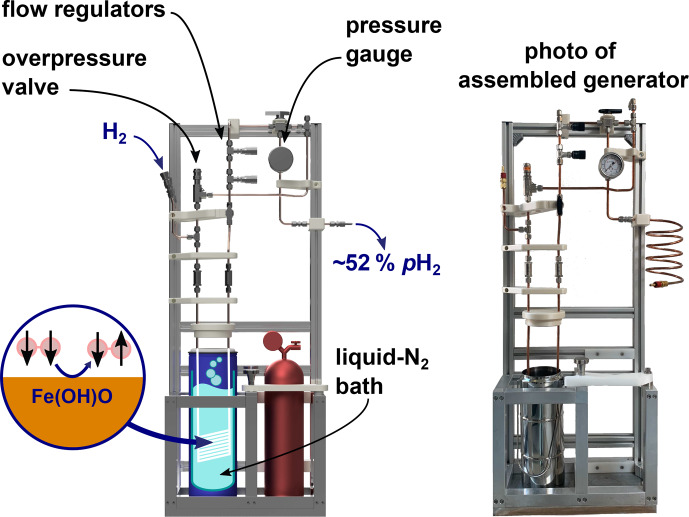
Rendering of the PHG (left) and a photo of the final build (right). The design of the PHG is open-source and simple and uses off-the-shelf as well as 3D-printed parts.

A PHG fulfilling the initial design requirements was successfully constructed (Fig. 6). Most parts were either commercially available, 3D-printed or simple to construct on-site. The holders for the bottles and a bottom plate were the sole parts prepared by a mechanical workshop. All parts were rated for more than 100 bar and no H ${}_{2}$ leaks were detected at 50 bar of H ${}_{2}$ using a H ${}_{2}$ detector (GasBadge Pro H2, Industrial Scientific, Pittsburgh, USA). Inspection and operation were facilitated by easy access and open construction design. The total cost was below EUR 3000 (Appendix A).

**Figure 7:**
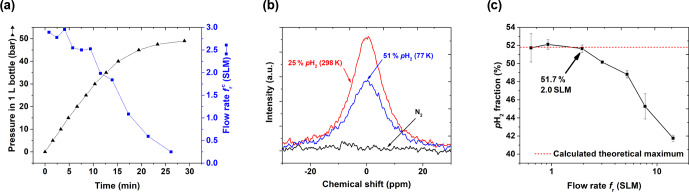
PHG operating parameters and NMR spectra: **(a)** pressure $p_{\mathrm{out}}$ and calculated flow rate $f_{r}^{c}=p_{\mathrm{out}}^{\prime }\cdot \frac{V_{0}T_{\mathrm{stand}}}{T_{\mathrm{rt}}P_{\mathrm{stand}}}$ as a function of time for input pressure $p_{\mathrm{in}}=50$ bar and $V_{0}=1$ L, **(b)** ${}^{1}$ H NMR spectra of rtH ${}_{2}$ , p H ${}_{2}$ and N ${}_{2}$ to quantify $f_{pH_{2}}$ , and **(c)** $f_{pH_{2}}$ as a function of $f_{r}$ (Eq. 3). For the latter, the para-enrichment was found to be constant up to a flow rate of $f_{r}=2$ SLM (for $p_{\mathrm{in}}=20$ bar, $p_{\mathrm{target}}=10$ bar).

We deliberately abstained from including a flow meter in the setup to keep the cost low and increase the robustness. Instead, we monitored the pressure $p_{\mathrm{out}}$ in the storage cylinder and calculated the flow rate (Fig. 7a). The expected increase in pressure and decrease in the flow rate of p H ${}_{2}$ were observed. The flow rate is an important parameter since it affects the number of H ${}_{2}$ collisions with the catalyst in the ortho-para-conversion unit (Figs. 3, A3) that enables fast para-ortho conversion. A lN ${}_{2}$ -based PHG can provide $f_{pH_{2}}≅52$ % at maximum (Fig. 2, 7b). If the flow rate is too fast, the gas will not have enough time to reach the ortho-para thermal equilibrium while passing through the unit. Hence the p H ${}_{2}$ fraction will be reduced.

Thus, to find optimal performance conditions of the PHG, we quantified $f_{pH_{2}}$ as a function of the flow rate (Fig. 7c) set by the needle valves (Fig. 3, A5). At the given settings of $p_{\mathrm{in}}=20$ bar and $p_{\mathrm{target}}=10$ bar, $f_{pH_{2}}\approx 51.7$ % was found for a flow up to $f_{r}=2$ SLM. For larger flow rates, the enrichment dropped significantly. Given these data and to allow for some variation, we chose a standard operating flow of ∼0.9 SLM. This flow rate was fast enough for convenient p H ${}_{2}$ production. For example, l L of 49 bar p H ${}_{2}$ with $f_{pH_{2}}=(51.7\pm 0.8$ ) % was produced in 29 min ( $p_{\mathrm{in}}=49$ bar, initial flow rate of 2.9 SLM, Fig. 7a).

### 3.2 The precision of p H ${}_{2}$ production, quantification and lifetime

**Figure 8:**
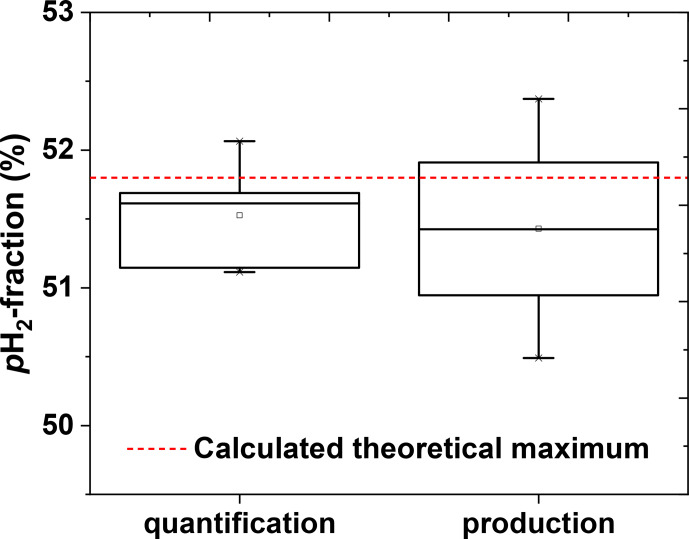
Reproducibility of the p H ${}_{2}$ quantification method (left) and p H ${}_{2}$ production cycle (right). The left boxplot shows the precision of the quantification method. The p H ${}_{2}$ quantification protocol was repeated five times with the same p H ${}_{2}$ batch. The obtained p H ${}_{2}$ fraction was ( 51.5±0.4 ) % that gives us an impression of quantification precision. The right boxplot shows the reproducibility of the production. The production of p H ${}_{2}$ and quantification protocols were repeated once on 4 different days. The obtained p H ${}_{2}$ fraction here was ( 51.6±0.9 ) %; the error value includes production and quantification errors. PHG parameters of p H ${}_{2}$ preparation: 20-bar inlet pressure, 10-bar final pressure in the storage cylinder and $f_{r}=0.9$ SLM. All errors are given by the standard deviation.

To test the reproducibility of the quantification method, $f_{pH_{2}}$ of a single batch was quantified five times in a row (including venting, flushing, and filling of the tube). The average $f_{pH_{2}}$ was found to be ( 51.5±0.4 ) %, corresponding to a coefficient of variation (CV) of 0.7 % (Fig. 8).

To access the reproducibility of the entire production process, four p H ${}_{2}$ batches were produced on different days and quantified. An average $f_{pH_{2}}$ of ( 51.6±0.9 ) % was observed (CV = 1.7 %) (Fig. 8).

We also investigated $f_{pH_{2}}$ as a function of the inlet pressure $p_{\mathrm{in}}$ at a fixed flow rate: a batch was prepared for $p_{\mathrm{in}}$ equal to 12, 20, 35, and 50 bar, $p_{\mathrm{target}}=10$ bar, and a flow rate of 0.9 SLM. No pressure dependency was observed. The obtained average of $f_{pH_{2}}$ was ( 52.4±0.8 ) %.

**Figure 9:**
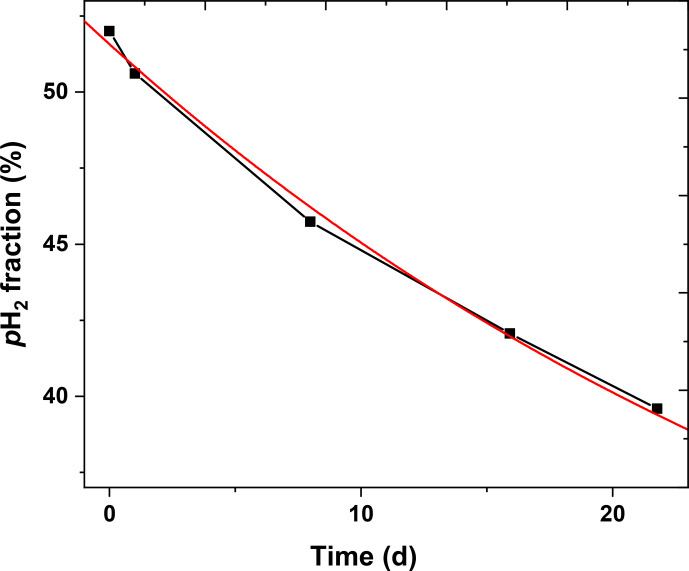
p H ${}_{2}$ lifetime in a 2 L aluminium cylinder. After production, five samples of p H ${}_{2}$ were taken from the cylinder (days 0, 1, 7, 15 and 21) and quantified (squares). An exponential function $A_{1}\cdot \exp(-t/\tau )$ + $y_{0}$ with $y_{0}=25$ (red line) was fitted to the data and yielded $A_{1}=26.6\pm 0.3$ and a decay constant of τ= ( 35.5±1.5 ) d.

To evaluate the lifetime of p H ${}_{2}$ in the 2 L cylinder, a 10-bar p H ${}_{2}$ batch was produced ( $p_{\mathrm{in}}=20$ bar, $f_{r}=0.9$ SLM). Over 22 d, five samples were taken from the batch and $f_{pH_{2}}$ was quantified. An exponential decay function was fitted to the data and yielded a constant of 35.5±1.5 d (Fig. 9).

### 3.3 Application: ${}^{1}$ H-low-field SABRE at different p H ${}_{2}$ pressures

**Figure 10:**
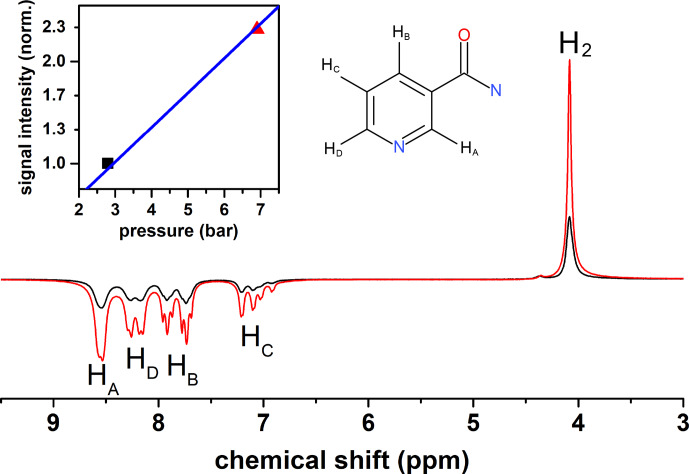
${}^{1}$ H-SABRE spectra of nicotine amide and H ${}_{2}$ at 6.9 bar and 2.8 bar p H ${}_{2}$ pressure. (Insert): signal intensity of nicotine amide vs. pressure with a linear fit (blue line). Nicotine amide structure is added for convenience.

The presented setup was designed to allow for pressures up to 50 bar. High pressures are beneficial for hyperpolarization because the concentration of p H ${}_{2}$ in the solution increases with pressure. A low concentration of p H ${}_{2}$ is often the limiting factor of the hyperpolarization payload (polarization level × concentration of polarized species). To demonstrate the effect, we polarized nicotine amide by SABRE and magnetic field cycling (scheme in Fig. 5) at two different p H ${}_{2}$ pressures: 2.8 and 6.9 bar (Fig. 10). Strong polarization was observed on the ${}^{1}$ H resonances of nicotine amide and hydrogen in solution. A 2.5-fold increase in pressure yielded a 2.3-fold increase in nicotine amide polarization.

## 4 Discussion

### 4.1 Design

The design of the presented PHG is simple and compact without compromising on performance and safety. The PHG is small and portable (although a heavy bottom plate was added for stability). Since there are no electrical components, it can be placed indoors as well as outdoors and does not require any electrical power supply. Note that electric components can be an ignition source which may lead to an explosion in case of a hydrogen leak.

For the framework, mostly off-the-shelf parts were used. More complex geometries, e.g. holders for valves or gauges, were 3D-printed. They have individual shapes and dimensions, and manufacturing in a workshop might lead to high costs and long production lead times. Three-dimensional printing turned out to be a versatile manufacturing method enabling fast prototyping, complex shapes, and low cost for one-off productions. The design of the PHG and all 3D models (STL files, Standard Triangulation Language, and CAD files, computer-aided design) are provided, enabling other groups to adjust the parts to their individual needs (Ellermann, 2020b).

Choosing a small 2 L dewar keeps the design compact and the running costs low since less than 2 L of liquid nitrogen was required to prepare 1 L of p H ${}_{2}$ at 50 bar. In combination with a short cooling down time, the setup is well suited for an on-site p H ${}_{2}$ production in a hyperpolarization lab.

#### 4.1.1 Costs

The final cost of the PHG incl. the hydrogen sensor was EUR 2988 incl. VAT (19 %). If a hydrogen sensor is already available in the lab, the overall costs for the PHG may be less than EUR 2500 incl. VAT (19 %). A complete set including the PHG, a hydrogen sensor, hydrogen/nitrogen gas as well as a variety of essential tools cost about EUR 3700 incl. VAT (19 %).

#### 4.1.2 Safety

All parts which are in contact with pressurized gas are rated to at least 100 bar. However, we fixed the operation pressure to 50 bar to get a generous safety margin of 100 %. The risk of static and inductive spark charges in the gas line is low (Department of Labour of New Zealand, 1990). Nevertheless, the gas pipes can be grounded to prevent electrical charges on the parts which are in contact with H ${}_{2}$ gas.

The design of the PHG incorporates a gas path which also enabled safe ventilation of a storage bottle. Potential handling errors were also considered. For example, the output connectors are closed for pressures up to 17 bar when they are disconnected, i.e. the case when the storage bottle is disconnected. Thus, there is no contact between the air in the room and the hydrogen in the PHG. Furthermore, we included a handheld hydrogen sensor that measures a hydrogen concentration as low as parts per million. The sensor should be always turned on during operation to indicate a potential leak of H ${}_{2}$ .

The setup includes low-temperature cryogens such as liquid nitrogen. To prevent the spilling of liquid nitrogen, the dewar was restrained by the copper tubing inside and the surrounding metal frame. Moreover, a lid covered the liquid nitrogen bath that also reduced the evaporation rate of the cryogen. Since the flask only holds around 2 L of cryogen, the amount of liquid nitrogen that has to be handled is greatly reduced.

Note that the PHG should be placed in a non-public restricted-access enclosure or room with sufficient ventilation and that only instructed personnel should operate it.

Our presented safety concept is economical and practical without sacrificing any safety measures. Nevertheless, after setting up the PHG, it should be tested for leakage with an inert gas or nitrogen. The part list also contains a leak detection spray and the H ${}_{2}$ sensor.

#### 4.1.3 Performance

The enrichment achieved here, e.g. $f_{pH_{2}}=51.6\;\%\pm 0.9$ % for $p_{\mathrm{in}}=20$ bar, $f_{r}=0.9$ SLM, was close to the maximum of 51.8 % conditioned by the boiling point of lN ${}_{2}$ and somewhat higher than reported elsewhere: $f_{pH_{2}}$ = 50 % (Barskiy et al., 2016a, 2016b; Shchepin et al., 2016). Determining the enrichment as a function of flow allowed us to choose an optimal flow of 0.9 SLM for $p_{\mathrm{in}}=20$ bar: this rate was e.g. sufficient to fill 1 L bottles to 10 bar in 10 min. The central design criterion of high pressure was successfully met as 1 L of 49 bar p H ${}_{2}$ was produced in 28 min ( $p_{\mathrm{in}}=50$ bar). We demonstrated that an increase in p H ${}_{2}$ pressure can give a proportional increase in polarization (Fig. 10). Obviously, this approach is limited as soon as the hyperpolarization yield is no longer determined by the availability of p H ${}_{2}$ and cannot provide a polarization above 33 % (Korchak et al., 2018).

#### 4.1.4 p H ${}_{2}$ quantification and production reliability

The automatic quantification process featured a CV of 0.7 %; the p H ${}_{2}$ production and quantification together featured a CV of 1.7 % (Fig. 8). These results indicate that the routine p H ${}_{2}$ quality control can be performed with a low-cost 1 T benchtop NMR spectrometer. The automatization certainly helped to make the process more reliable but was not necessary. Feng et al. (2012) used the similar quantification approach and reported a precision of 1 %–3 % for quantification. NMR is a convenient method for p H ${}_{2}$ quantification, but optical methods may be used, too (Parrott et al., 2019).

#### 4.1.5 The lifetime in aluminium cylinder

The relaxation time constant in aluminium tanks was found to be ( 63.7±8.3 ) d by Feng et al. (2012) and ∼120 d by Hövener et al. (2013), respectively. We found here a shorter lifetime of ( 35.5±1.5 ) d in our 2 L aluminium storage bottle. Note that we did not perform any dedicated cleaning procedure for the p H ${}_{2}$ storage bottle. Still, the lifetime was sufficiently long to produce p H ${}_{2}$ once a week.

## 5 Conclusions

The presented PHG enables the production of p H ${}_{2}$ with $f_{pH_{2}}\approx 52$ % at a high pressure of 50 bar reliably (CV = 1.7 %), providing about one-third of the polarization achieved with $f_{pH_{2}}$ ≈ 100 %. Because the device delivers high-pressure p H ${}_{2}$ , however, this effect can be partially compensated in the PHIP/SABRE experiment. A new, automated quantification routine at 1 T benchtop NMR proved to be reliable and simple (CV = 0.7 %). The design of the PHG is straightforward and easy to manufacture with openly available blueprints and at a cost of less than EUR 3000. The device may facilitate further research on the promising method of parahydrogen-based hyperpolarization.

## Data Availability

All experimental data (SpinSolve H NMR spectra of H and blueprints for the PHG will be available from figshare.com (DOI: https://doi.org/10.6084/m9.figshare.13176830) (Ellermann et al., 2020a). Additionally, all blueprints are also accessible via git ((https://gitlab.tardis.rad.uni-kiel.de/fellermann/opensource-liquid-n2-based-ph2-generator) (Ellermann et al., 2020b).

## Appendix A

**Table A1:** Price list of all needed components for the high-pressure PHG. Full list of items required for construction of the portable liquid nitrogen parahydrogen generator (without cart) with 6 mm copper connection tubes. Here the following format for item names is used: {Name (company), (specifications), article number}. Given prices include 19 % VAT.

			Price, incl.	Price, incl.
No.	Name	Amount	VAT (Euro/unit)	VAT (Euro)
Gases and cylinders (EUR 148.00)				
1	Hydrogen cylinder (Air Liquide), (gas, ALPHAGAZ1, S10-1.8 m3), P0231S10R2A001	1	30.00	30.00
2	Nitrogen cylinder (Air Liquide), (gas, ALPHAGAZ1, S10-1.8 m3), P0271S10R2A001	1	40.00	40.00
3	Aluminium cylinder (Luxfer), (cylinder, H ${}_{2}$ valve 28,8/21,8 LH), A6341Q	1	78.00	78.00
Regulators, gauges, and valves (EUR 1578.92)				
4	Hydrogen cylinder pressure regulator (Air Liquide), (EUROJET 200/50, DIN 477-1, 6 mm tube outlet), Eurojet_125607	1	279.00	279.00
5	Nitrogen cylinder pressure regulator (Kayser), (in 200 bar, out 0–20 bar, DIN 477-1, 1/4 ${}^{\prime \prime }$ mm male ISO parallel thread), CK1302	1	128.00	128.00
6	Stainless steel one-piece 40G series ball valve (Swagelok), 0.6 Cv, 6 mm tube fitting	1	124.12	124.12
7	Stainless steel high-pressure proportional relief valve (Swagelok), 6 mm tube fitting	1	253.83	253.83
8	Purple spring for proportional relief valve (Swagelok), 750 to 1500 psig (51.7 to 103 bar)	1	6.90	6.90
9	Stainless steel one-piece 40G series three-way ball valve (Swagelok), 0.90 Cv, 6 mm tube fitting	1	191.00	191.00
10	Stainless steel low-flow metering valve (Swagelok), 6 mm tube fitting, vernier handle	2	242.52	485.04
11	Gauge up to 100 bar (Swagelok) (6 mm tube fitting)	1	111.03	111.03
Tube and fittings (EUR 346.15)				
12	Copper tube, CUR6X1R Kupferrohr 6×1 mm, R 220, rolled good, soft, 1 m (Landefeld)	5	11.29	56.45
13	Brass instrumentation quick-connect stem with valve (Swagelok) 0.2 Cv, 6 mm tubefitting	2	27.01	54.03
14	Brass instrumentation quick-connect body (Swagelok) 0.2 Cv, 6 mm bulkhead tube fitting	2	54.43	108.86
15	Stainless steel tube fitting (Swagelok), Union, 6 mm tube OD	1	17.91	17.91
16	Stainless steel Swagelok tube fitting, Union Tee, 6 mm tube OD	3	36.30	108.90
Rest (catalyst, H ${}_{2}$ detector, dewar, ..., EUR 1278.20)				
17	Fe(OH)O (Merck) (catalyst grade, 30–50 mesh), 371254-50G	1	152.00	152.00
18	Hydrogen detector (Industrial Scientific) (GasBadge Pro H2, 0–2000 ppm), 18100060-C	1	499.00	499.00
19	Stainless steel liquid nitrogen dewar flask (isotherm) (DSS 2000, 2L, h = 305, D = 114), 2103	1	413.00	413.00
20	Stainless steel in-line particulate filter (Swagelok), 6 mm tube fitting, 90 micron pore size	2	107.10	214.20
Gas exhaust line (EUR 59.80)				
21	Polyamide tube (AS-Drucklufttechnik GmbH) ( 6×3 mm, 1 m), PA 6X3 BLAU-25	5	1.36	6.80
22	Multiple plug connection (AS-Drucklufttechnik GmbH) ( 1×8 mm, 4×6 mm), IQSQ 8060	1	16.00	16.00
23	Reduction plug nipple (AS-Drucklufttechnik GmbH) (8 to 6 mm), IQSG 80H60	1	5.90	5.90
24	Check valve (AS-Drucklufttechnik GmbH) (6 mm, 0.2 opening < 0.2 bar), HIQS 60	1	31.10	31.10

**Table A1:** Continued.

			Price, incl.	Price, incl.
No.	Name	Amount	VAT (Euro/unit)	VAT (Euro)
Tools (EUR 125.00)				
25	Clip (AS-Drucklufttechnik GmbH) (0–14 mm), SAS 14	1	9.2	9.20
26	Leak detection spray (HM INDUSTRIESERVICE GMBH), 291-1252	1	15.1	15.10
27	Adjustable wrench RF 300 (Proxxon), (max 34 mm), 23994	1	26	26.00
28	Open-ended wrench set	1	16.2	16.20
29	Pipe cutter (Landefeld), (3–30 mm), 4333097000773	1	57.5	57.50
30	PTFE thread seal tape	1	1.00	1.00
Frame parts (EUR 132.17)				
31	Rexroth profile 2 m each ( 2× 980 mm, 5×340 mm)	2	38.28	76.56
32	Rexroth *Nutenstein* M4/M5	14	1.12	15.66
33	Filament for 3D printer: Ultimaker PLA *Perlweiß* 2.85 mm 750 g	1	39.95	39.95
34	In-house-built metal parts (e.g. from university workshop)	0	0	0
35	Screws M4/M5	0	0	0
Total price for the basic equipment, incl. hydrogen sensor, excl. VAT				2511.12 ${}^{*}$
Total price for the basic equipment, incl. 19 % VAT				**2988.24** ${}^{*}$
Total price for the complete set, incl. tools and gases, excl. VAT				3082.55*
Total price for the complete set, incl. tools and gases, incl. 19 % VAT				3668.24 ${}^{*}$

${}^{*}$ Overall costs may vary due to change in prices and change in VAT rate and due to costs which may arise for custom parts (e.g. material or labour costs from the facility's workshop).

## Code availability

Software for automatic signal recording and gas control will be available from figshare.com (DOI: https://doi.org/10.6084/m9.figshare.13176830, Ellermann et al., 2020a) and via git ((https://gitlab.tardis.rad.uni-kiel.de/fellermann/opensource-liquid-n2-based-ph2-generator) (Ellermann et al., 2020b).
